# Case report: Immunoadsorption therapy for anti-caspr1 antibody-associated nodopathy

**DOI:** 10.3389/fimmu.2022.986018

**Published:** 2022-09-21

**Authors:** Lili Liu, Juanjuan Chen, Yue Zhang, Jun Wu, Jun Hu, Zhijian Lin

**Affiliations:** ^1^ Department of Neurology, Peking University Shenzhen Hospital, Shenzhen, China; ^2^ Department of Nephrology, Peking University Shenzhen Hospital, Shenzhen, China

**Keywords:** anti-Caspr1 antibody, CIDP, nerve biopsy, nodo-paranodopathies, IA therapy

## Abstract

**Background and objectives:**

Several autoantibodies against proteins located at the node of Ranvier has been identified in patients with chronic inflammatory demyelinating polyneuropathy (CIDP) in the last few years. Then a new concept, autoimmune nodo-paranodopathies was proposed. Cases of Caspr1 autoantibodies are the most rare. Here we describe an anti-Caspr1 nodopathy patient, summarized his clinical, physiological and pathological features.

**Case presentation:**

We present the case of a 56-year-old male patient with proprioceptive loss, ataxia, coarse tremor and distal limb weakness without any painess and cranial involvement. Electrophysiological studies showed prolonged distal motor latencies, conduction slowing and reduced amplitude distal compound muscle action potential (CMAP) amplitude. Antibodies against the nodes of Ranvier in serum samples revealed a positive finding for the anti-Caspr1 antibody (1:10).Myelinated fiber loss could be revealed in nerve biopsy. Longitudinal ultrathin sections of the nodal region was discovered in electron microscope, the paranodal/nodal architecture was destructed. It was lack of transverse bands and enlargement of the space between the axon and the paranodal loops was seen. The patient improved obviously after three times immunoadsorption(IA) therapy.

**Conclusion:**

Anti-Caspr1 nodopathy patient may present atypical symptoms without any neuropathic pain and cranial palsy. The destruction of paranodal/nodal architecture could be observed in nerve biopsy, which may be caused by the lost of axoglial complex formed by NF155, CNTN1 and Caspr1. Antibodies detection is important for the diagnosis, while IA therapy could be regarded as an option for the patients allergic to rituximab (RTX).

## Introduction

Several autoantibodies against proteins located at nodes of Ranvier have been identified in patients with chronic inflammatory demyelinating polyneuropathy (CIDP) in the last few years (1). These autoantibodies include anti-neurofascin, anti-contactin1 (CNTN1), and anti-contactin-associated protein 1 (Caspr1) antibodies that predominantly belong to the IgG4 subclass (2). They do not activate complement- or cell-mediated cytotoxicity, but have high affinity for their antigens that results in disruption of axoglial interactions (3, 4). Patients with these antibodies have a common phenotype characterized by advanced age, aggressive onset, severe axonal damage, tremor, ataxia, and poor response to intravenous immunoglobulin (IVIG) (5). Paranodal dissection without classical demyelination is the characteristic pathological feature (6). Thus, a new concept, autoimmune nodo-paranodopathies, was proposed (7).

Caspr1 is a cell-adhesion protein that form a complex with CNTN1. This complex binds to the glial protein NF155 to form septate-like junctions that maintain ion channel clustering at nodes of Ranvier (8, 9). Patients with autoantibodies against Caspr1 have the same symptoms as those with other nodo-paranodopathies. Demyelination was detected in nerve conduction studies, while axonal degeneration was observed in nerve biopsies (10).

The first-line treatment options for CIDP are corticosteroids, IVIG, and plasma exchange (PE) (11), while patients with autoantibodies against node of Ranvier proteins may be treated with rituximab (12). For patients with plasma or RTX allergy, immunoadsorption (IA) therapy would be a safe option. Selective IA is considered a suitable alternative to classical PE, because it has similar efficacy and safety to PE but avoids substitution with human plasma products. Immediate intravascular reduction of autoantibody concentrations, pulsed induction of antibody redistribution, and immunomodulatory changes are the major mechanisms for IA therapy (13, 14).

Here we describe an anti-Caspr1 antibody-associated nodopathy patient who presented with proprioceptive loss, ataxia, coarse tremor, and distal limb weakness without any pain, and showed a good response to IA therapy. This case expands our understanding of anti-Caspr1 antibody-associated nodopathies and provides evidence for IA therapy in these diseases.

## Materials and methods

### Case presentation

**Figure 1:**
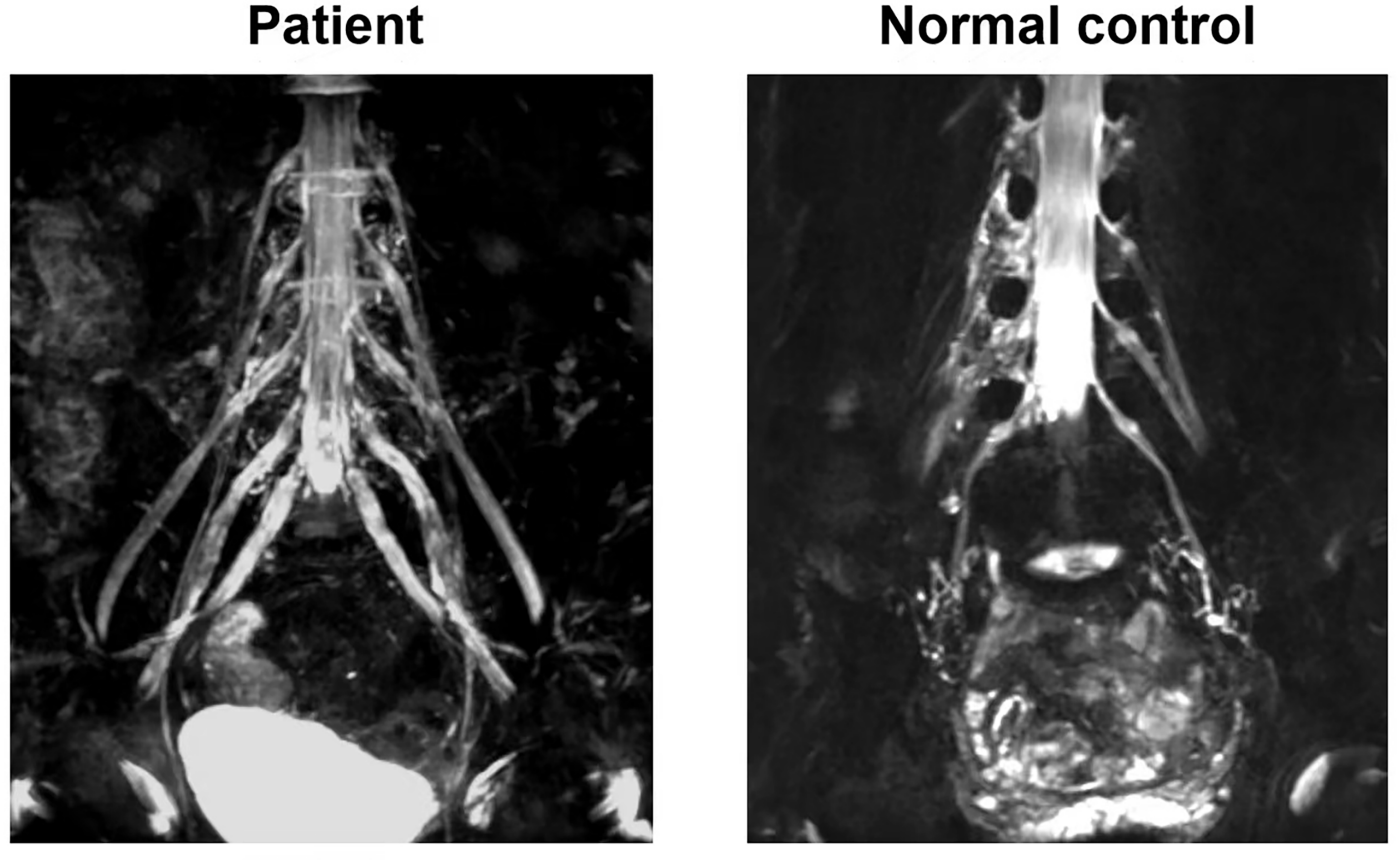
MRI neurography with enhancement imaging of lumbosacral spinal roots and plexuses. The patient showed marked symmetric hypertrophy and increased signal intensity in lumbosacral roots and plexuses comparing with normal control.

A 56-year-old man was admitted in our hospital because of progressive limb paresthesias and weakness for 8 weeks. His symptoms began subacutely with numbness in the feet and fingers, and then gradually progressed proximally to involve the knees and hands. He was treated with vitamins B1 and B12 at a clinic and showed a mild response. In the previous 2 weeks, his symptoms had aggressively developed with weakness and ataxia. He exhibited foot-drop, steppage gait, and difficulty in walking in a straight line. His medical history included hypertension and hypertriglyceridemia. Neurological examination at admission revealed normal cranial nerve function, motor weakness (4/5) affecting the distal lower extremities, hyporeflexia, decreased proprioception, and superficial sensation in a glove-and-stocking pattern. Accuracy in the finger-to-nose and heel-to-knee-to-shin tests, tremor, and positive Romberg’s sign were noted. His Overall Neuropathy Limitations Scale (ONLS) score was 5. The neuroelectrophysiological results were as follows: obvious prolongation of motor distal latency in the bilateral peroneal, tibial, and median nerves; marked reduction of motor conduction velocity in the bilateral medianus, ulnar nerve, bilateral tibialis, and peroneus; ≥30% reduction in the proximal relative to distal negative peak compound muscle action potential (CMAP) amplitude in the bilateral tibialis and peroneus; there was no response to stimulation in sensory conduction of the medianus, ulnaris, radial nerve, peroneus, and suralis. The F wave conduction velocity of the bilateral medianus was markedly decreased. The occurrence rate of F waves in the lower extremities was decreased less than 30% with a prolonged latency (**Supplement 1**). The protein level in the cerebrospinal fluid (CSF) was severely elevated at 2.68 g/L. Other CSF tests, routine blood tests, biochemical tests, and serum electrophoresis were negative. Tests for paraneoplastic antibodies(Hu, Yo,Ri, CV2/CRMP5, Ma1, Ma2, SOX1, Tr, Zic4, GAD65, PKCg) and anti-ganglioside antibodies (GM1, GM2, GM3, GM4, GD1a, GD1b, GD2, GD3, GT1a, GT1b, GQ1b) in the serum were negative in the serum and CSF yielded negative results. Magnetic resonance imaging (MRI) neurography with enhancement was conducted using a 3.0-T whole-body clinical imager (Ingenia; PHILIPS, The Netherlands). The patient showed marked symmetric hypertrophy and increased signal intensity in the lumbosacral roots and plexuses (**Figure 1**).

### Cell-based assays for antibodies against NF155, NF186, CNTN1, and Caspr1

Antibodies against NF155, CNTN1, NF186, and Caspr1 were detected by immunocytochemistry using human embryonic kidney 293 (HEK293) cells transfected with the respective plasmids of interest. HEK293 were plated onto poly-L-lysine coated glass coverslips in 24-well plates at a density of 50 000 cells/wells and were transiently transfected with NF155, NF186,CNTN1 and Caspr1 constructs (NM.001160331, NM.001005388, NM.001843, NM.003632) using pcDNA3.1-co-expressed green fluorescent protein(GFP) in lipofectamine 2000 (Thermo Fisher). HEK293 cells were maintained in Dulbecco/Vogt modified Eagle’s minimal essential medium supplemented with 10% fetal bovine serum, 1% l-glutamine, and 1% penicillin/streptomycin. At 24 hours after transfection, the cells were fixed with 4% paraformaldehyde for 15 minutes and permeabilized with 0.3% Triton X-100 for 5 minutes. The cells were then incubated with the patient’s serum (1:10) or CSF (1:1) preabsorbed with an HEK293 cell pellet for 1h at room temperature, followed by incubation with corresponding orange fluorescently-labeled secondary goat against human IgG antibodies(Beijing, Biosynthesis Biotechnology Co., Ltd) 1.5h at room temperature. The labeled cells were mounted with Fluoromount™ medium (Sigma-Aldrich). Images were acquired using a fluorescence microscope (Model IX73; Olympus).

### Nerve biopsy and histological tissue processing

Sural nerve biopsy and tissue preparation procedures were performed using a standard protocol (15). The nerve was cut into three parts: the first was embedded in paraffin for hematoxylin and eosin, myelin basic protein, and neurofilament staining; the second was embedded in Epon, cut into transverse semithin (1-mm) sections, and stained with toluidine blue for nerve fiber examination under an optical microscope(Leica DM 2500 LED); the third was cut into ultrathin (90-nm) sections, and stained with lead citrate and uranyl acetate for electron microscopic observation(JEOL 1011 electron microscope) (16).

## Results

### Nerve biopsy and immunohistochemistry findings

**Figure 2:**
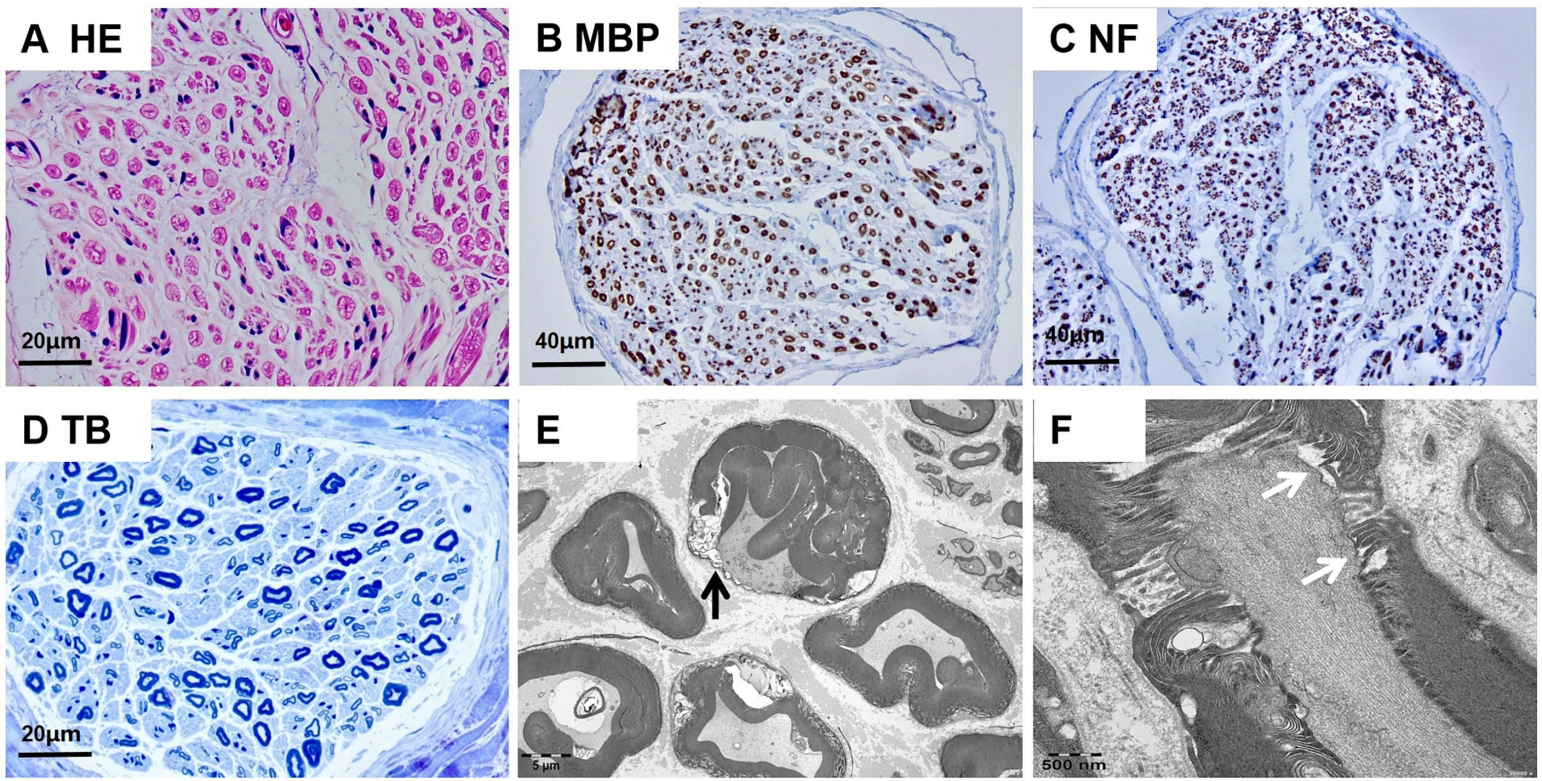
Neuropathologic findings of sural nerve biopsy. **(A-D)** Optical microscopy showed no presentation of demyelinated fibers, infiltration of inflammatory cells, or onion bulb formation. **(E)** Transmission electron microscopy showed demyelination(black arrow), and no axonal degeration was observed. **(F)** Longitudinal ultrathin sections of the nodal region showed the enlargement of the space between the axon and the paranodal loops(white arrow). Scale bars = 20 µm **(A, D)**; 40 µm **(B, C)**; 5 µm **(E)**; 500nm **(F)**.

Demyelinated fibers, infiltrated inflammatory cells, and onion bulb formation were not detected on microscopic observation (**Figures 2A–C**). Myelinated fiber loss was noted, but was not severe (**Figure 2D**). Transmission electron microscopy showed demyelination (black arrow), and no axonal degeration was observed (**Figure 2E**). Electron microscopic observation of longitudinal ultrathin sections of the nodal region revealed destruction of the paranodal/nodal architecture. Enlargement of the space between the axons and paranodal loops was seen (**Figure 2F**).

### Detection of autoantibodies against paranodal proteins

**Figure 3:**
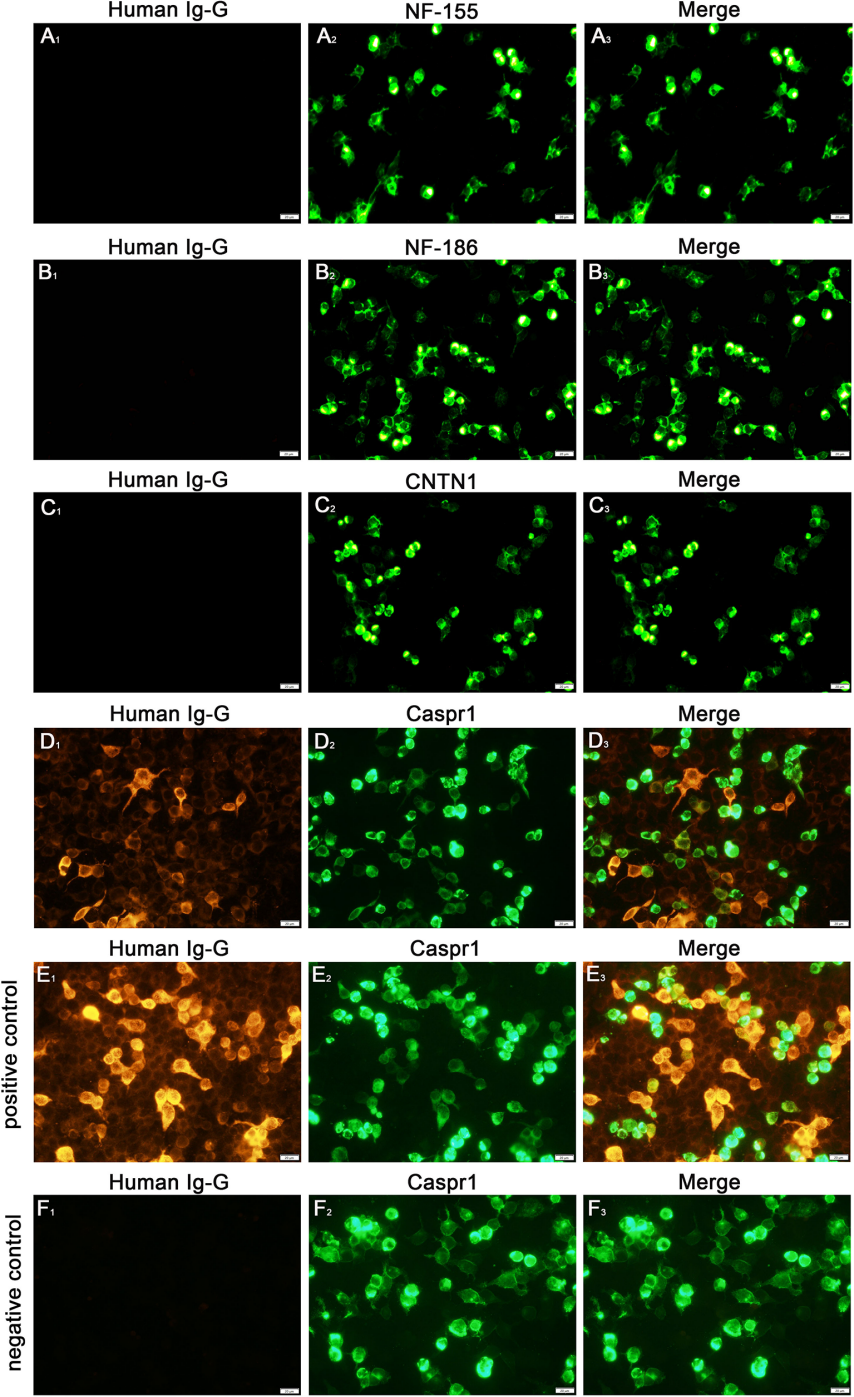
Detection of anti-NF155, NF186, CNTN1 and Caspr1 antibodies **(A-D)**. Cell-based assays revealed a positive finding for the anti-Caspr1 antibody (1:10) in serum **(D)**, the **(E, F)** were the positive and negative control of the anti-Caspr1 antibody. The positive control group used the serum of patient with positive anti-Caspr1 antibody and the negative control group used the serum of patient with negative anti-Caspr1 antibody. All the patients’ serum (1:10) were preabsorbed with an HEK293 cell pellet for 1h at room temperature, followed by incubation with corresponding orange fluorescently-labeled secondary goat against human IgG antibodies(Beijing, Biosynthesis Biotechnology Co., Ltd) 1.5h at room temperature. Scale bar = 20 µm.

Analysis for autoantibodies against node of Ranvier proteins in his serum samples revealed positive findings for anti-Caspr1 antibodies (1:10) and negative findings for anti-NF155, anti-NF186, and anti-CNTN1 antibodies. we did not identify the isotype for the patient in our study (**Figure 3**).

### IA therapy and outcomes

Based on the clinical manifestations, auxiliary examination, nerve biopsy findings, and antibody examination, the patient was diagnosed as anti-Caspr1 antibody-associated paranodopathy. The patient was administered high-dose methylprednisolone (1 g/day) for 5 days, but showed poor improvement. Because he was allergic to rituximab, adjunctive IA therapy for protein A was conducted. The plasma was first separated from the blood taken out of the body by a plasma separator (blood flow: 100 to 150 ml/min). The separated plasma was then passed on to staphylococcal protein A immunoadsorption column, KONPIA^®^ (KCIA08, Guangzhou KONCEN Biotechnology Co., LTD) by a plasma pump at a flow rate of 30-40 mL/min for 15-20 minutes of adsorption. After adsorption was completed, the bypass was opened, and normal saline was supplied for plasma transfusion back to the patient at a flow rate of 70 mL/min. When the color of the plasma inflow tube turned pale, the adsorbed antibodies were eluted with eluent at a flow rate of 70 mL/min. A pH of 2-2.8 indicated the completion of elution. The solution was subsequently neutralized to pH 7.0 with equilibrium liquid and pre-flushed again with normal saline in order to complete the regeneration of adsorption column. After the adsorption column was regenerated, the plasma could be re-extracted for re-adsorption. Five-to-ten consecutive cycles of adsorption constitute one treatment course. Plasma volume of 500-600mL can be treated in a single adsorption cycle. The total treated plasma volume can reach 3000-6000 mL. The patient underwent IA therapy three times on alternate days. The plasma separator and tubing system were single-use only, but the IA adsorber for protein A was used for three sessions before the expiration date.

The patient became able to walk independently after receiving IA therapy three times. His ONLS score was 2 at hospital discharge(the 25th day), and had returned to zero at the 2-month follow-up.

## Discussion

CIDP is a progressive immune disease of the peripheral nerves. Although patients with antibodies against nodal-paranodal cell adhesion molecules (CNTN1, NF155, Caspr1, NF140/186) fulfil the 2010 European Academy of Neurology/Peripheral Nerve Society criteria for CIDP (17), the revised guideline in 2021 proposed to classify the diseases as “autoimmune nodopathies” and to not regard them as CIDP variants (18). Presence of autoantibodies has been demonstrated in approximately 1.9%–5.5% of patients with CIDP (1, 12, 19, 20), although some cohorts had rates of almost 20% (12, 20). These discrepancies may due to differences in the study populations, and the incidence may be higher in Asia than in Europe. Anti-Caspr1 antibody-associated nodopathies are particularly rare, and their frequency was estimated at 2.9% in a small German study (8). Up to now, only four cases have been reported in China (including the present case), and thus larger studies are needed to determine the accurate incidence in the future.

Anti-Caspr1 antibody associated nodopathy patients are mostly middle-aged, with acute or subacute onset. In previous reports, neuropathic pain accompanied by cold sensation was the characteristic clinical manifestation. Very recently, antibodies targeting the Caspr1/CNTN1 complex were discovered (10, 21). Rapid onset with cranial nerve involvement was considered a clinical clue for this subtype. In our Chinese case, the patient had obvious proprioceptive loss, ataxia, coarse tremor, and distal limb weakness, without any neuropathic pain or cranial palsy, similar to the findings in Italian patients (12). It has been suggested that anti-Caspr1 or anti-Caspr1/CNTN1 complex antibody-associated nodopathies may comprise a spectrum of disorders, with patients presenting different combinations of symptoms and severities that depend on ethnic group, age, sex, and IgG isotype, but the underlying mechanism remains to be established. Compared with seronegative CIDP patients, the ONLS score was higher in patients with antibodies against node of Ranvier proteins, and motor dysfunction was more pronounced in the lower extremities than in the upper extremities (22, 23), consistent with the features in our patient. The main causes of the disabilities in our patient were the severe ataxia and distal weakness of the feet, which differed greatly from typical CIDP.

Laboratory data for anti-Caspr1 antibody-positive CIDP patients showed greatly increased CSF protein level and prominent gadolinium enhancement in the nerve roots on MRI neurography (8, 10, 12, 20, 24), similar to the case for our patient. These findings suggest the presence of inflammatory reactions and blood-nerve barrier disruption in the nerve roots and plexus, such that antibodies may pass through these sites to travel to nodes of Ranvier (25). The electrophysiological examination in our patient showed prolonged distal motor latencies, conduction slowing, and reduced distal CMAP amplitude, indicating demyelination and axonal involvement. Some acute-onset nodopathy patients also showed conduction slowing, but it promptly resolved without development of remyelination characteristics in electrophysiological examinations. This rapid recovery of conduction slowing was named reversible conduction failure (RCF), and is considered to arise from a temporary dysfunction at nodes of Ranvier. Therefore, serial electrophysiological tests can help to increase the diagnostic accuracy, and detection of RCF may suggest that the lesion site in the nerve is at a node of Ranvier, which can be recovered quickly with a good prognosis (5, 26).

Because autoantibodies have become useful biomarkers for diagnosis and treatment selection, their detection is important in clinical practice. In the early stage, peptide-based ELISA and western blotting analyses were used for antibody assays, but were found to have low specificity (3, 5), mainly because the presented antigens were not in their native conformation. Meanwhile, a cell-based assay (CBA) can present a more natural antigen conformation, whereby the protein of interest is expressed by transfected cells, and thus the revised guideline recommends CBA use as an antibody screening test. In our patient, to avoid false-positive results and achieve highly-specific testing, we conducted a CBA antibody detection.In our patient, destruction of the paranodal/nodal architecture was obvious, and enlargement of the space between the axons and paranodal loops was detected by electron microscopy. These ultrastructural alterations suggested a lesion in the paranodal region and myelin loop detachment, which may have been caused by loss of the axoglial complex formed by NF155, CNTN1, and Caspr1 (5, 6). Myelin loop detachment and increased periaxonal space may reduce the paranodal transverse resistance, increase the time necessary to depolarize the next node producing slow conduction, and fail to transmit the impulse to the next node. These considerations can explain the electrophysiological demyelination features observed in nodopathies.

The first-line treatments for CIDP are corticosteroids, IVIG, and PE, while RTX is recommended in seropositive CIDP patients, especially those with paranodal antibodies (11, 12). Poor response to IVIG in nodopathy patients was observed in a prospective clinical trial, and was explained by the lack of complement fixation capacity of IgG4 subtype antibodies (27). In our case, the patient was resistant to steroids and allergic to RTX, but exhibited a prominent response to IA therapy. The therapeutic effects of IA in autoimmune diseases can be attributed to immediate intravascular reduction of the autoantibody concentration, and subsequent immunomodulatory changes. But Kuwahara W. found that the tryptophan column used for IA is suitable for the IgG1 and IgG3, and was not effective in patient with NF155-IgG4 subtype antibodies. So we speculated that the patient in our study may have non-IgG4 subtype antibodies (28). Since only small sample has been reported, the therapeutic effect of IA on different subtypes antibodies of nodopathy needs further study. Comparing to plasma exchange, IA seems to have a favorable side effect spectrum, but immunoglobulin levels reduction, which may increase the risk of severe infections, were reported in some observational studies (29). In our study, no adverse events was observed after 2-month follow-up. To our knowledge, this is the first case of IA therapy for an anti-Caspr1 antibody-associated nodopathy patient, and our findings demonstrate that IA therapy can be regarded as an effective and safe option for this disease.

The following limitations of the study have to be mentioned: we did not identify the antibody isotypes, and differences in the antibody isotypes may be a reason for the disparities in the clinical manifestations. It is also very important to make a clinical strategies. Furthermore, the levels of anti-Caspr1 antibodies after treatment were not evaluated, which would objectively evaluate the effects after IA therapy.

In conclusion, an anti-Caspr1 antibody-associated nodopathy patient may present with proprioceptive loss, ataxia, coarse tremor, and distal limb weakness without any neuropathic pain or cranial palsy. Destruction of the paranodal/nodal architecture and enlargement of the space between the axon and paranodal loops can be observed in nerve biopsy findings. Antibody detection is important for the diagnosis, and IA therapy can be regarded as an option for patients who are allergic to rituximab.

## Data availability statement

The original contributions presented in the study are included in the article/**Supplementary Material**. Further inquiries can be directed to the corresponding author.

## Ethics statement

Written informed consent was obtained from the individual(s), and minor(s)’ legal guardian/next of kin, for the publication of any potentially identifiable images or data included in this article.

## Author contributions

LL wrote the manuscript, performed literature retrieval and utilization, contributed to the figure and table preparation, and helped in the diagnostic process. YZ performed the immunoadsorption. JW, JH, and ZL were involved in the care of the patient. JC performed the nerve biopsy, assisted in the diagnostic process, supported the interpretation, and critically revised the manuscript. All authors contributed to the article and approved the submitted version.

## Acknowledgments

The authors thank Alison Sherwin, PhD, from Liwen Bianji (Edanz) (www.liwenbianji.cn/) for editing the English text of a draft of this manuscript.

## Conflict of interest

The authors declare that the research was conducted in the absence of any commercial or financial relationships that could be construed as a potential conflict of interest.

## Publisher’s note

All claims expressed in this article are solely those of the authors and do not necessarily represent those of their affiliated organizations, or those of the publisher, the editors and the reviewers. Any product that may be evaluated in this article, or claim that may be made by its manufacturer, is not guaranteed or endorsed by the publisher.

## Supplementary material

Supplement 1 Dynamic changes in electromyography (EMG).
