# Supplementary material for: Case report: Immunoadsorption therapy for anti-caspr1 antibody-associated nodopathy
Source: Front Immunol. 2022 Sep 21;13:986018. doi: 10.3389/fimmu.2022.986018 (PMC9532626; doi:10.3389/fimmu.2022.986018)
Supplement: Supplement 1 — Dynamic changes in electromyography (EMG). [file Table_1.docx]

Supplement1

|  | Normal | Case prsentation(L/R) |
| --- | --- | --- |
| Median nerve |  |  |
| Distal latency(ms) | <4 | 8.1/7.5 |
| MCV(m/s) | >50 | 29/25 |
| CAMP amplitude(mv) | >8 | 5.8/4.9 |
| F wave latency(ms) | >50 | 67.4/50 |
| F wave frequency | >73% | 93%/90% |
| Ulnar nerve |  |  |
| Distal latency(ms) | <3 | 5.2/4.8 |
| MCV(m/s) | >50 | 26/24 |
| CAMP amplitude(mv) | >7 | 6.5/4.8 |
| F wave latency(ms) | >50 | 73/69 |
| F wave frequency | >73% | 91%/85% |
| Tibial nerve |  |  |
| Distal latency(ms) | <5 | 11.3/15.1 |
| MCV(m/s) | >45 | 24/21 |
| CAMP amplitude(mv) | >9 | 0.6/0.4 |
| F wave latency(ms) | <51 | 137.2/112 |
